# Effects of bevacizumab plus irinotecan on response and survival in patients with recurrent malignant glioma: a systematic review and survival-gain analysis

**DOI:** 10.1186/1471-2407-10-252

**Published:** 2010-06-02

**Authors:** Tao Xu, Juxiang Chen, Yicheng Lu, Johannes EA Wolff

**Affiliations:** 1 Department of Neurosurgery, Changzheng Hospital, Second Military Medical University, Shanghai, 200003, China; 2 Departments of Pediatrics and Biostatistics, The University of Texas M. D. Anderson Cancer Center, Houston, TX, USA

## Abstract

**Background:**

The combination of bevacizumab and irinotecan is a new chemotherapy protocol increasingly used for recurrent malignant glioma. Results from phase II trials suggest this drug combination is beneficial to patients, but no conclusive comparisons between this and other treatment protocols have been published.

**Methods:**

We performed a systematic review and survival gain analysis of phase II studies to evaluate the efficacy and safety of bevacizumab plus irinotecan treatment. To do this, we utilized a preexisting database from which the mean overall survival and response rate of patients could be predicted. Survival gain, which characterized the influence of treatment, was defined as the difference between observed and predicted mean overall survival. Response gain was calculated similarly.

**Results:**

741 cohorts were enrolled in the database. Among them, 282 cohorts were based on recurrent adult HGG, mean reported median overall survival was 10.96 ± 8.4 months, and mean response rate was 18.9% ± 20.5. We found that compared with other treatment protocols, bevacizumab plus irinotecan largely improved response rates (*P *= 0.00002) and had a possible moderate effect on overall survival time (*P *= 0.024). Hemorrhage, thromboembolic complications, and gastrointestinal toxicities were the most frequently reported side effects.

**Conclusion:**

The combination of bevacizumab and irinotecan might improve outcome in patients with recurrent malignant glioma. Randomized controlled trials are recommended to evaluate this treatment protocol and the additional value of irinotecan.

## Background

High-grade glioma (HGG), also named malignant glioma, is the most common brain tumor in adults, and the outcome of patients with HGG remains poor [1]. The standard of care for adult patients with glioblastoma is radiation and temozolomide [2]. However, this regimen yields median survival times of only 12 to 15 months for patients with newly diagnosed glioblastomas and only 2 to 5 years for patients with newly diagnosed anaplastic gliomas [3]. Once the tumors recur the prognosis is even worse, with a median survival of only 3 to 9 months regardless of the treatment regimen [4,5]. Very few evidence-based treatment options are available for patients with recurrent disease, and treatment response for glioblastoma has generally been less than 20%, with a 6-month progression-free survival (PFS) rate of less than 9-16% [4,6]. One of the difficulties in finding other effective treatment regimens is the variability of outcome data in HGG studies, which is, at least in part, caused by disease heterogeneity and differing eligibility criteria [7,8].

Topoisomerase 1 inhibitors, such as irinotecan and topotecan, provide a viable treatment option for tumors resistant to temozolomide since the mechanisms of action of these two classes of drugs and the known mechanisms of resistance do not overlap [9]. Irinotecan was proved to have activity against non-glioma malignancies, such as gastrointestinal malignancies [10]. It was also regarded as an alternative choice for recurrent HGG despite the controversy regarding its ability to pass through the blood brain barrier. However, as a single agent, irinotecan showed disappointing results in the treatment of recurrent malignant gliomas [11].

Bevacizumab, the humanized monoclonal antibody against vascular endothelial growth factor (VEGF), has been approved by the U. S. Food and Drug Administration (FDA) for the treatment of colorectal, lung, and breast cancers. In May 2009, FDA has granted accelerated approval for single bevacizumab for use in patients with glioblastoma that has progressed despite previous therapy. Bevacizumab has generally been used in combination with cytotoxic agents [12,13]. However, the value of combining bevacizumab with irinotecan to treat HGG is still unclear. The treatment response rates ranged from 28 to 86%, with a 6-month PFS ranging from 9.5 to 78.6%. With this large variation in outcome data the question of the effectiveness of this drug combination remains open.

Recently, a phase II study survival gain meta-analysis was reported using novel mathematical methods to compare different nitrosourea drugs [8]. Here we did a systematic review of published phase II trials of bevacizumab plus irinotecan for recurrent HGG and used the previous mathematical technology to analyze the survival and response benefit of this treatment protocol.

## Methods

### Identification and selection of studies

This analysis was based upon a database that had been created for a treatment arm summarizing analyses by compiling information on HGGs from literature published from 1976 to 2008. This database had been used previously in comparing different nitrosourea drugs [7,8]. This method generally falls under the umbrella of meta-analysis [14]. The preexisting database was expanded through May 2008.

In order to address the question of our study a further independent search was carried out by querying PubMed (updated through September 2009), EMBASE (1980-September 2009), and Cochrane controlled-trials registry databases using the search words "bevacizumab," "irinotecan," "CPT-11," "glioma," and "glioblastoma." No language or date limitations were imposed. The following selection criteria were applied: (1) Study population of patients with histologically proven malignant glioma, all of whom had experienced tumor progression that was measurable on magnetic resonance imaging (MRI) and received bevacizumab plus irinotecan as salvage chemotherapy; (2) Study contained information on the diagnosis of recurrent malignant glioma, treatment protocol, criteria for response, responses to treatment, and overall survival or PFS; (3) The response rates for recurrent HGG were measured by two consecutive MRI scans and the Macdonald criteria were used. The partial response was regarded if the contrasted MRI showed more than 50% decrease in the area of enhancement and stable or decreased T2 and FLAIR signal, with a stable or decreased dose of corticosteroid and a stable or improved clinical status. The complete response was determined by the resolution of all measurable abnormalities on the contrast images, as well as by stable or decreased disease on T2 and FLAIR images for any patient who was on a stable or decreased corticosteroid dose and with stable and improved clinical status. Disease progression was regarded if there was more than 25% increase in the area of enhancement, appearance of a new lesion, or deterioration in the patient's clinical status that was thought to be related to tumor progression. The patient was deemed to be stable if the criteria for a partial or complete response or tumor progression were not met and if there was no disease progression. (4) In case of duplicate publication of the same patient cohort, only the most recent publication was used for further analyses. The decision to include a trial was made separately by two of our researchers (Xu and Chen), who then compared their lists and resolved any discrepancies.

### Data extraction

As described previously, the analysis was based upon a database. Briefly, patient cohorts were generated into a database based on every published paper about HGG. Each patient cohort included more than 30 parameters, such as general characteristics (mean age, sex, etc.), pathological diagnosis, treatment response, and survival time. If there was more than one group of patients included in one paper, patient cohorts were generated separately depending on the different groups. The median overall survival time was recorded from each study. Other outcome parameters and population characteristics were analyzed in relation to median overall survival and then used to calculate predicted median overall survival using various multiple regression models; no treatment information was used in calculating the predicted overall survival and response. Finally, the predicted median overall survival was compared to the reported median overall survival time and the difference was called "survival gain". Response was quantified in the same way as overall survival. The influence on response of parameters characterizing eligibility criteria and patient cohort was analyzed and used to compute the predicted response rate (complete response and partial response) of a given patient cohort. The "response gain" was then compared between groups.

To analyze the effect of the combination of irinotecan and bevacizumab on median overall survival and treatment response, comparisons were made between recurrent HGG patient cohorts treated with both drugs and those treated with neither. Histograms were created for visual comparison, and the Mann-Whitney *U *test was applied when appropriate.

All analyses were done using SPSS software, version 16.0. (SPSS Inc^® ^Fulfillment center Haverhill MA, SPSS 16.0) and P < 0.05 was regarded as statistical significant.

## Results

### Overall survival of the database

**Figure 1:**
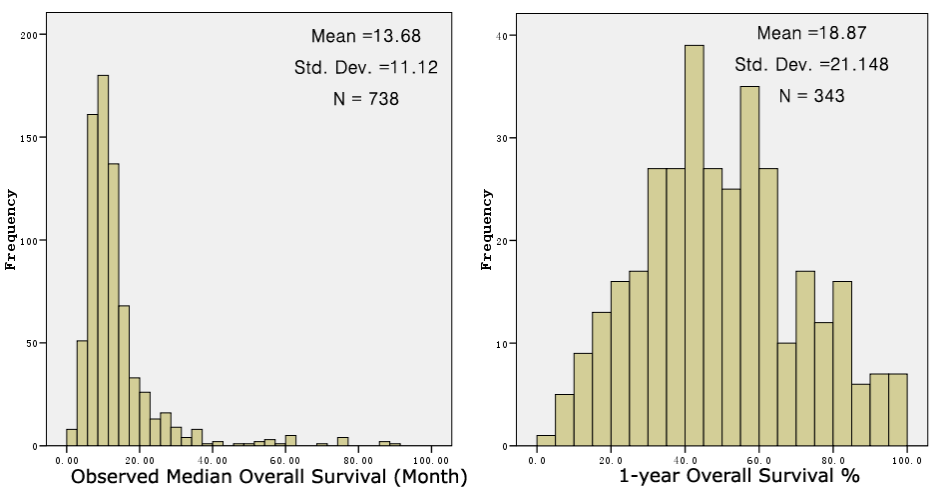
**Observed median overall survival and percentage of 1-year overall survival for patients with malignant glioma in all published data**. These data showed a median overall survival time of 13.7 ± 11.1 months and 1-year overall survival percentage of 18.9 ± 21.1% for malignant glioma patients.

The database contained 547 cohorts between 1973 and 2008 when it was used in last published paper, with a mean reported median overall survival of all cohorts being 13.7 months (standard deviation [SD], 11.7 months) [8]. Now that the database had been expanded from 547 to 741 cohorts, the mean reported median overall survival time increased to 13.7 months (SD 11.1 months), and 1-year overall survival percentage was 18.9 ± 21.1% (Fig. 1). Among the 741 cohorts, 282 cohorts were based on recurrent adult HGG, with a mean of median survival time of 10.96 months (SD 8.4 months).

### Response rates of the database

**Figure 2:**
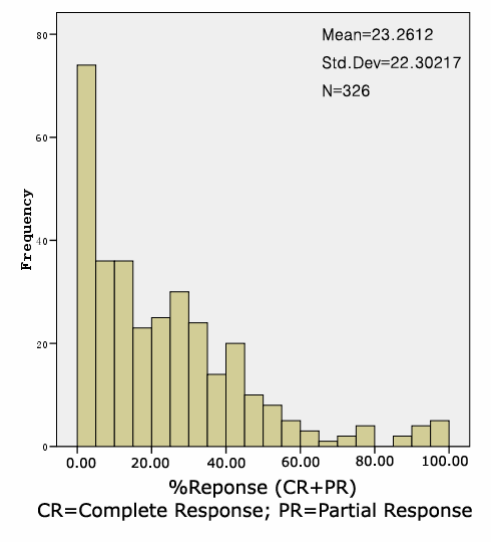
**Distribution of treatment response rates for patients with malignant glioma in all published data**. The mean response rate was 23.3 ± 22.3% in 326 studies.

Among the 326 cohorts that reported response rates, the mean response rate was 23.3% (range 0-100; see Fig. 2 for distribution). The response rate was significantly different when patients with newly diagnosed HGG were compared with patients with recurrent HGG (32.6% ± 23.4 versus 18.9% ± 20.5), when patients with glioblastoma only were compared with patients with anaplastic astrocytoma (18.2% ± 17.2 versus 30.8% ± 24.9).

### Description of studies with irinotecan and bevacizumab

A total of 12 studies met all our inclusion criteria, and 10 of the 12 studies were enrolled in the survival gain-analysis [15-24]. The other two studies were excluded because of duplicate patient cohorts [25,26]. The basic characteristics of the enrolled cohorts are described in Table 1, and the treatment response is listed in Table 2.

**Table 1:** Basic characteristics of enrolled studies

Study	**No**.	Gender (n)		Median age (year)	Histology (n)		Treatment
					
		Male	Female		WHO IV	WHO III	
Chen [15]	21	11	10	58	17	4	Beva (10 mg/kg) + irino (340 mg/m^2 ^for EIAED; 125 mg/m^2 ^for non-EIAED) every other week

Vredenburgh[16]	35	22	13	48	35	0	Beva (10 mg/kg) + irino (340 mg/m^2 ^for EIAED; 125 mg/m^2 ^for non-EIAED) Q14d, beva (15 mg/kg) Q21d + irino (340 mg/m^2 ^for EIAED, 125 mg/m^2 ^for non-EIAED) on days 1,8,22, and 29, on a 6-week cycle

Bokstein [17]	20	14	6	56	17	3	Beva (5 mg/kg) + irino (125 mg/m^2^) every other week

Guiu [18]	77	50	27	52	49	28	Beva (10 mg/kg) + irino (340 mg/m^2 ^for EIAED; 125 mg/m^2 ^for non-EIAED)

Ali [19]	13	7	6	53	13	0	Beva (5 mg/m^2^) every 2 weeks + irino (125 mg/m^2^) every week for 3 weeks with 1 week off; beva (10 mg/m^2^) + irino (125-250 mg/m^2^) every 2 weeks

Desjardins [20]	33	22	11	43	0	33	Beva (10 mg/kg) + irino (340 mg/m^2 ^for EIAED; 125 mg/m^2 ^for non-EIAED) Q14d, beva (15 mg/kg) Q21d + irino (340 mg/m^2 ^for EIAED, 125 mg/m^2 ^for non-EIAED) on days 1,8,22, and 29, on a 6-week cycle

Kang [21]	27	NR	NR	46	12	15	Beva (10 mg/kg) + irino (340 mg/m^2 ^for EIAED; 125 mg/m^2 ^for non-EIAED) on days 1 and 15 every 28 days

Poulsen [22]	52	34	18	46	28	24	Beva (10 mg/kg) + irino (340 mg/m^2 ^for EIAED; 125 mg/m^2 ^for non-EIAED) every 2 weeks

Zuniga cohort A [23]	14	9	5	51	0	14	Beva (10 mg/kg) IV on days 1,15,29 for each cycle; irino (340 mg/m^2 ^for EIAED; 125 mg/m^2 ^for non-EIAED) every 2 weeks, on a 6-week cycle

Zuniga cohort B [23]	37	24	13	53	37	0	Beva (10 mg/kg) IV on days 1,15,29 for each cycle; irino (340 mg/m^2 ^for EIAED; 125 mg/m^2 ^for non-EIAED) every 2 weeks, on a 6-week cycle

Friedman [24]	82	57	25	57	82	0	Beva (10 mg/kg) + irino (340 mg/m^2 ^for EIAED; 125 mg/m^2 ^for non-EIAED) every 2 weeks

*No*. number of patients, *Beva *bevacizumab, *Irino *irinotecan, *EIAED *enzyme-inducing antiepileptic drugs, *Q14d *every 14 days, *Q21d *every 21days

**Table 2:** Treatment response of bevacizumab plus irinotecan for patients with recurrent malignant glioma

Study	mPFS	PFS 6-month (%)	mOS	OS 6-month	Response rate (%)	Survival gain	Response gain
Chen [15]	2.4	9.5	8.5	61.9	38	4.23	21.37

Vredenburgh [16]	5.5	46	9.6	77	57	5.07	42.18

Bokstein [17]	4.2	25	7	55	47	0.73	31.02

Guiu [18]	NR	NR	NR	NR	36	-7.91	22.72

Ali [19]	5.5	46.2	6.2	53.85	77	2.81	61.96

Desjardins [20]	6.9	55	14.9	79	61	4.27	36.38

Kang [21]	5.1	45.8	12.6	84	44	2.94	24.34

Poulsen [22]	5	32.4	6.9	NR	28	-0.65	8.24

Zuniga Cohort A [23]	13.4	78.6	NR	85.7	86	12.6	61.49

Zuniga Cohort B [23]	7.6	63.7	11.5	78	76	6.72	60.71

Friedman [24]	5.6	50.5	8.7	NR	37.8	1.24	22.84

*mPFS *median progression-free survival, *mOS *median overall survival

Response rate (%) = complete response (%) + partial response (%)

NR = not reported

411 patients were included in our analysis. The median progression-free survival time ranged from 2.4 to 13.4 months, the median overall survival time ranged from 6.2 to 14.9 months, with response rates ranging from 28% to 86%. Different doses of bevacizumab were used in these studies, but most patients received 10 mg/kg, while some other patients received 5 mg/kg, 5 mg/m^2 ^and 15 mg/kg. The dose of irinotecan was mainly stable; most patients received 340 mg/m2 for those who take enzyme-inducing antiepileptic drugs (EIAED) or 125 mg/m2 for those with non-EIAED. In each cohort there was no statistical difference between the results of patients receiving EIAED or non-EIAED patients. Similarly, in all included studies there were no statistical differences between the results for patients receiving different cycles of bevacizumab plus irinotecan.	

### Toxicity

Toxicity and side effects of bevacizumab plus irinotecan for patients with recurrent malignant glioma are listed in Table 3. Hemorrhage, thromboembolic complications (e.g., thrombotic thrombocytopenic purpura, deep venous thrombosis, pulmonary embolism, myocardial infarction), and gastrointestinal toxicities (e.g., diarrhea, gastrointestinal perforation) were most frequently reported. Further side effects included renal dysfunction (proteinuria, hematuria), fatigue, and neutropenia.

**Table 3:** Toxicity of bevacizumab plus irinotecan for patients with recurrent malignant glioma

Study	Number of patients	Toxicity or side effects	
Chen [15]	21	Not reported	
Vredenburgh [16]	35	Thromboembolic complications (n = 4), Grade II proteinuria (n = 2), Grade II fatigue and withdrew consent(n = 4), Grade III or worse gastrointestinal toxicity (n = 4), Sepsis (n = 1), central nervous system hemorrhage (n = 1), leg ulcers (n = 1)	
Bokstein [17]	20	Grade I hypertension (n = 1), Grade I rash (n = 1), Grade I and III fatigue (n = 3), Grade II epistaxis (n = 1), Grade II anemia (n = 1), Grade II diarrhea (n = 2), Grade II seizures (n = 1), Grade III paranoid psychosis (n = 1)	
Guiu [18]	77	Intratumoral hemorrhage (n = 5, with spontaneous regression in 3) and thromboembolic complications including venous thrombophlebitis (n = 4), pulmonary embolism (n = 2), and myocardial infarction (n = 1); Grade III-IV hepatotoxicity (n = 2), reversible leukoencephalopathy (n = 1).	
Ali [19]	13	Intracranial bleeding (n = 2), deep venous thrombosis (n = 1)	
Desjardins [20]	33	Dose reduction due to gastrointestinal toxicity or neutropenia (n = 5), withdrew consent due to fatigue or gastrointestinal toxicity (n = 4), central nervous system hemorrhage (n = 1), thrombotic thrombocytopenic purpura (n = 1)	
Kang [21]	27	Thromboembolic complications (n = 5), hemorrhage (n = 3), hematuria (n = 1), infection (n = 1), fatigue (n = 1), cough (n = 1), failure to thrive (n = 1)	
Poulsen [22]	52	Grade III cerebral hemorrhage (n = 1), cardiac arrhythmia (atrial fibrillation), intestinal perforation (n = 1), superficial venous thrombosis (n = 1), hypertension (n = 3), neutropenia (n = 1), infection (n = 2), proteinuria (n = 1); Grade V diarrhea (n = 1)	
Zuniga cohort A [23]	14	Grade II-III hypertension (n = 13), Grade I-II bleeding (n = 9)	____
Zuniga cohort B [23]	37		Grade III proteinuria-renal failure (n = 1), gastrointestinal perforation (n = 1), severe nausea/vomiting (n = 4)
Friedman [24]	79	Hypertension (n = 21; Grade≥3 hypertension: n = 1); Hemorrhage, overall (n = 32; Grade≥3: n = 2); Hemorrhage, intracranial (n = 3; Grade≥3: n = 1); Wound-healing complications (n = 2; Grade≥3: n = 1); Venous thromboembolism (n = 8; Grade≥3: n = 7); Arterial thromboembolism (n = 5, Grade≥3: n = 2); Proteinuria (n = 2; Grade≥3: n = 1); Gastrointestinal perforation (n = 2; Grade≥3: n = 2); Reversible posterior leukoencephalopathy syndrome (n = 1, Grade≥3: n = 0); Aphasia (Grade≥3: n = 6); Confusional state (Grade≥3: n = 4); Convulsion (Grade≥3: n = 11); Diarrhea (Grade≥3: n = 4); Fatigue (Grade≥3: n = 7); Pneumonia (Grade≥3: n = 4); Pyramidal tract syndrome (Grade≥3: n = 4); Somnolence (Grade≥3: n = 4); Hypokalemia (Grade≥3: n = 6); Leukopenia (Grade≥3: n = 5); Lympyhopenia (Grade≥3: n = 6); Neutropenia (Grade≥3: n = 7)	

### Comparing bevacizumab plus irinotecan outcome with other HGG data

We compared the response gain and survival gain of recurrent HGG patients between the bevacizumab plus irinotecan cohorts (combination cohorts) and other treatment cohorts (control cohorts) using the Mann-Whitney *U *test.

**Figure 3:**
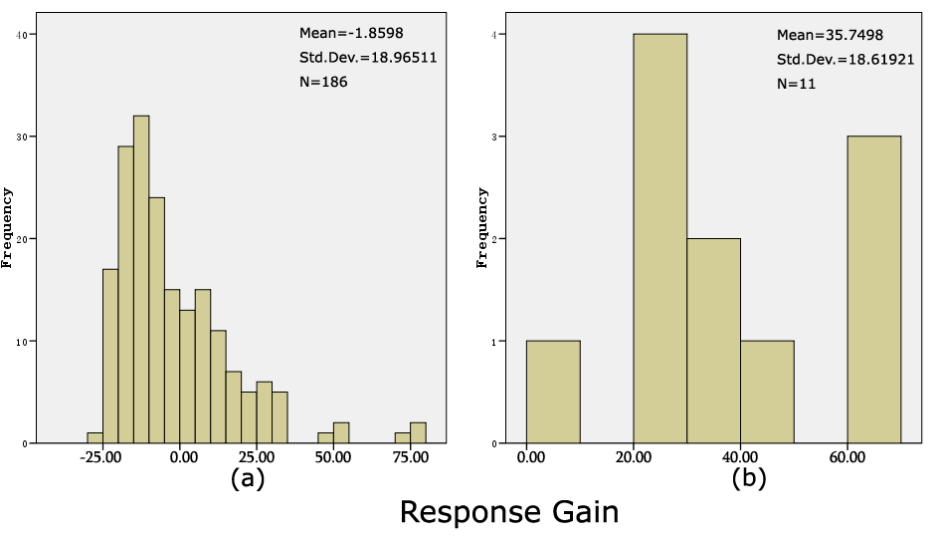
**Response gain distribution in control group (a) and bevacizumab plus irinotecan group (b)**. The combination of bevacizumab and irinotecan showed benefit in treatment response for patients with recurrent HGG.

We first compared the response gain of recurrent HGG patients treated with the combination protocol to those treated with other protocols and there was a clear result in favor of the novel drug combination: the mean rank of 186 control cohorts was 94.31, while the mean rank of 10 bevacizumab plus irinotecan cohorts was 178.27 (P = 0.00002). These results showed that the improved response rate after treatment with the combination of irinotecan and bevacizumab was highly statistically significant. The response gain distributions of the two groups are shown in Fig. 3.

**Figure 4:**
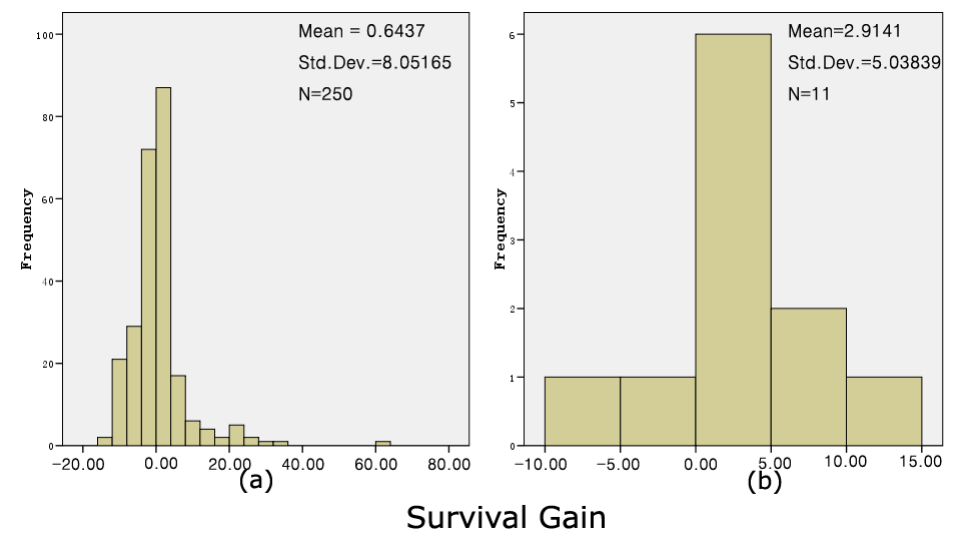
**Survival gain distribution in control group (a) and bevacizumab plus irinotecan group (b)**. The combination of bevacizumab and irinotecan showed benefit in survival time for patients with recurrent HGG.

We then tested the survival gain between recurrent HGG patients treated with bevacizumab plus irinotecan and those treated with other protocols. The Mann-Whitney *U *test showed that the mean rank of the 11 combination cohorts was 181.36, while the mean rank of 250 recurrent control cohorts was 128.78 (P = 0.024). The survival gain distributions of the two groups are shown in Fig. 4. There was an attempt to determine PFS as an additional endpoint, but because it was reported only rarely in the past the data of the control group were insufficient for the comparison (data not shown). In summary these results suggest that combination of bevacizumab and irinotecan can provide additional survival benefit compared to other treatment protocols in recurrent HGG patients.

## Discussion

The efficacy of bevacizumab plus irinotecan for recurrent malignant glioma has been evaluated in several clinical trials in recent years. However, the differences in patient characteristics made it difficult to judge the outcome results. Thus, we carried out a meta-analysis of the available evidence from phase II trials in order to more precisely define the efficacy of the combination. We found the treatment protocol might improve both the response rate and survival time for patients with recurrent malignant glioma, results that were consistent with the experience of others.

Response rate and overall survival are considered the two most important parameters in assessing the efficacy of any treatment protocol, yet one of the two is necessarily only a surrogate parameter. It is anticipated that treatment protocols that result in higher response rates will also result in higher survival rates in following phase III studies. In our analysis, the benefit of the drug combination appeared large when considering response, while the effect on overall survival was only marginal. However, response may translate directly into quality of life, and the fact that the two endpoints did not correlate strongly supports the concept that the response rate is an important additional parameter in evaluating efficacy of treatment protocols.

Anti-angiogenic treatment has been promising in the treatment of recurrent malignant gliomas [27,28]; so the Progression Free Survival time (PFS) was regarded as another important parameter for assessing efficacy of treatment protocols. However, a recent study carried out by Norden et al. found that PFS might not be an optimal endpoint for anti-angiogenic treatment [29] because the use of contrast-enhancement MRI may overestimate the response rates [30]. Anti-VEGF treatment can reduce vascular permeability, which can also account for the radiographic improvement; this may not necessarily reflect tumor cell death [31]. Decreased enhancement could be because of both tumor cell death and the anti-VEGF effect; thus, a more precise radiological measurement for treatment response is needed. Chen et al [15] reported ^18^F-fluorothymidine PET scanning could be used as an imaging biomarker to predict overall survival in patients with recurrent gliomas who are treated with the combined bevacizumab and irinotecan protocol. Other possibilities for measuring response include the entire FLAIR signal abnormality in T2 weighted MRIs or nuclear medicine methods such as thallium [32], amino acids [33], or glucose [34]. However, even with these measurements, the clinical relevance of these findings still remains a question.

Our finding that the response rate of an anti-angiogenic treatment may result in additional survival benefit is consistent with previous phase II studies. However, Norden et al. found that, compared to cytotoxic agents, anti-angiogenic therapy may fail to prolong overall survival in patients with recurrent malignant glioma [29]. It is also noteworthy that, according to the discrepancies between PFS and overall survival benefits, most patients died soon after disease progression when bevacizumab failed indicating that heavily pretreated patients may develop VEGF-independent mechanisms of progression and must be closely monitored [29].

The role of irinotecan in the treatment regimen has been argued [35]. Single agent irinotecan used to treat patients with recurrent malignant glioma did not show good results [11,36]. When irinotecan was added to bevacizumab response rates and overall survival seemed to improve in phase II trials. The reason for this is still unclear; one possibility is that the use of bevacizumab could decrease interstitial pressure, improve tissue oxygenation, and increase delivery of irinotecan to the tumor [37,38]. However, a recent study showed that adding irinotecan to bevacizumab did not result in additional improvement in overall survival time when compared with single-agent bevacizumab (overall survival, 8.9 months for combination and 9.7 months for single-agent) [39]. In our study, we did not compare survival gain and response gain between single-agent and combined treatment because of insufficient data for patients with recurrent malignant glioma treated with single-agent bevacizumab. Further prospective studies may provide more evidence to determine the additional role of irinotecan in the treatment of recurrent malignant glioma.

The toxicity of this treatment protocol should also be considered. In several meta-analyses, bevacizumab was reported to be associated with many side-effects, including venous thromboembolism [40], bleeding [41] and gastrointestinal perforation [42]. According to the clinical studies, hemorrhage, thromboembolic complications and gastrointestinal toxicities were most frequently reported (Table 3). The dose of bevacizumab may be related to vascular complications. In our analysis, compared with most studies that used the dose of 10 mg/m^2 ^for bevacizumab, Bokstein et al. used a dose of 5 mg/kg, which resulted in significantly lower vascular complication rates [17]. The gastrointestinal side effects were mainly due to irinotecan; these side effects may decrease the quality of life in these patients [43] and may even cause death [22].

The drug combination of irinotecan and bevacizumab resulted in responses on radiological imaging which can translate into increased survival. Also, this drug combination had some moderate toxicity. The question of whether the benefits of the drug combination outweighs the loss of quality of life caused by drug toxicity should be considered by each patient, and our analysis might help the doctors and patients to make a better decision.

Our study also had some limitations. First, all included studies were phase II studies. Thus, it might bring great heterogeneity because of the lack of control groups. Although patients who received other treatment protocols were set as control groups in this analysis, we still cannot draw a firm conclusion with these data. Larger phase III randomized controlled studies comparing bevacizumab plus irinotecan with other treatment protocols are warranted so that the efficacy can be assessed properly. Second, the difference of survival gain between groups (p value of 0.024) might be due to chance, although it was statistically significant. Compared with a p value of 0.00002 in response gain, it made us question whether the improved response really translated into a survival benefit for patients. Larger studies may be needed to answer this question. Third, we did not include the PFS-gain because of insufficient data. Although the role of PFS in anti-angiogenic treatment is still not clear, it is a very important parameter to assess the efficacy of treatment protocols. It was used as an endpoint in all 11 bevacizumab plus irinotecan cohorts of 10 studies. However, it was not used widely in early studies, and consequently cannot be calculated at the present time. Further analysis with more data might be helpful in this field.

## Conclusions

Our systematic review and survival gain analysis of phase II studies showed the drug combination of bevacizumab and irinotecan might improve overall survival and response rate in patients with HGG. Future randomized studies are warranted to evaluate this treatment protocol more precisely; the additional value of irinotecan as an addition to bevacizumab should also be considered in such studies.

## Abbreviations

HGG: High-grade glioma; PFS: Progression-free survival; VEGF: Vascular endothelial growth factor; FDA: Food and Drug Administration; MRI: Magnetic resonance imaging; SD: Standard deviation; EIAED: Enzyme-inducing antiepileptic drugs.

## Competing interests

The authors declare that they have no competing interests.

## Authors' contributions

XT and CJX carried out the search of studies, participated in the statistical analysis and drafted the manuscript. LYC participated in the design of the study and revised the manuscript. JEAW performed the statistical analysis, participated in its design, coordination and helped to draft the manuscript. All authors read and approved the final manuscript.

## Pre-publication history

The pre-publication history for this paper can be accessed here:

http://www.biomedcentral.com/1471-2407/10/252/prepub
